# A model of optical pump-terahertz probe by ultrafast terahertz near-field microscopy

**DOI:** 10.1016/j.isci.2026.115145

**Published:** 2026-02-25

**Authors:** Zijian Zhang, Ziyu Huang, Aojie Xu, Jing Li, Peiyan Li, Jiahua Cai, Mingcong Dai, Tianxiao Nie, Amine El Moutaouakil, Xiaojun Wu

**Affiliations:** 1 Hangzhou International Innovation Institute, Beihang University, Hangzhou 311115, China; 2 School of Electronic and Information Engineering, Beihang University, Beijing 100191, China; 3 Fert Beijing Institute, MIIT Key Laboratory of Spintronics, School of Integrated Circuit Science and Engineering, Beihang University, Beijing 100191, China; 4 Electrical and Communication Engineering, College of Engineering, UAE University, Al Ain, P.O. Box 15551, United Arab Emirates; 5 Zhangjiang Laboratory, Shanghai Advanced Research Institute, Chinese Academy of Sciences, Shanghai 201204, China

**Keywords:** Photonics, Radiation physics, Applied sciences

## Abstract

Terahertz scattering-type scanning near-field optical microscopy (THz s-SNOM) has emerged as a powerful tool for capturing information beyond the diffraction limit. Adding an external pumping laser enables nanoscale optical pump-THz probe (OPTP) spectroscopy, extending ultrafast dynamics studies to the nanoscale. However, unlike the thin-film approximation in far-field transmission, the relation between transient permittivity and the near-field OPTP signal remains computationally demanding to solve. Here, we introduce a generalized evolutionary model (GEM) derived from the physical independence between the ultrafast pump delay and the mechanical tip oscillation. By assuming a multiplicative evolution of the spectral response, GEM establishes a direct analytical link that decouples the transient permittivity from the complex near-field scattering integral. This approach allows for the rapid prediction of permittivity evolution without the need for full-time-domain spectral acquisition at every delay step. Our findings demonstrate that GEM significantly accelerates the extraction of transient permittivity during the pumping process, as validated on typical semiconductor samples.

## Highlights

• Our method allows rapid permittivity retrieval from near-field OPTP signals
• No need for full-time-domain spectral acquisition at every delay step
• Validated on SRAM, GaAs, and Bi2Te3 with high accuracy and 25x speedup

## Graphical abstract

**Figure:**
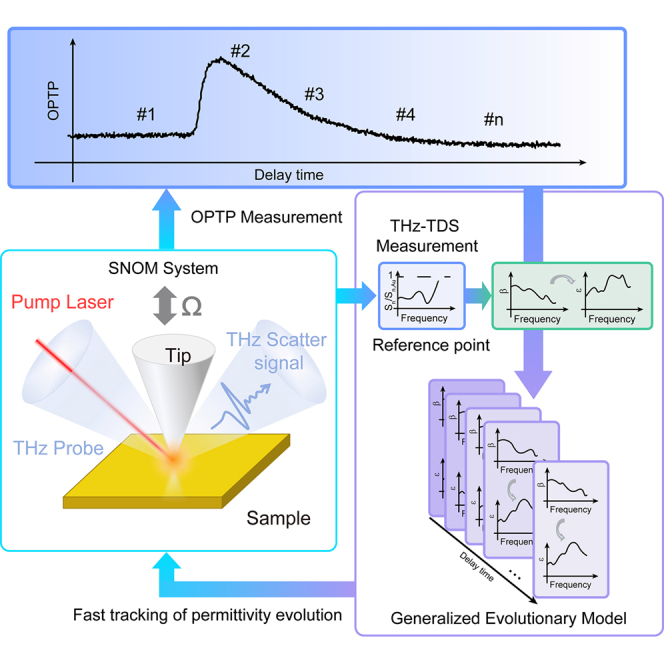

## Introduction

Terahertz-scattering-type scanning near-field optical microscopy (THz s-SNOM) is considered a highly effective technique for material characterization to exceed the diffraction limit.^1^^,^^2^^,^^3^ The minimum spatial resolution of the THz s-SNOM is determined by the radius of the tip of the atomic force microscope (AFM)^4^ This spatial resolution property allows researchers to observe nanoscale features using THz waves non-invasively. Thus, numerous applications of s-SNOM have been developed with the nanoscale resolution,^5^ including real-space imaging of polaritons,^6^ carrier imaging in transistor,^7^ the observation of phase-changing in vanadium dioxide,^8^ and extracting the permittivity locally.^9^^,^^10^ For a THz s-SNOM, the tip-sample interaction process can be expressed in finite dipole model (FDM) or point dipole model (PDM) models, where the AFM tip is treated as a dielectric sphere or ellipsoid to access the local permittivity of materials.^11^

**Figure 1:**
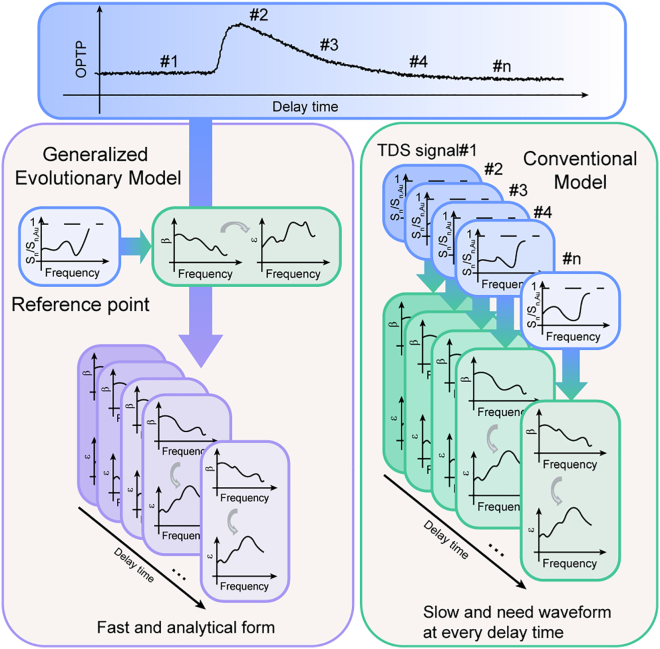
Schematic diagram of our GEM method vs. the conventional method Our proposed method (GEM) only needs an individual waveform and an OPTP signal, which are both easy to obtain. The usual method needs to capture a full waveform at every time delay, which is hard to realize in a short time.

Furthermore, researchers have combined THz s-SNOM with ultrafast pumping technology,^12^ successfully realizing Ultrafast THz s-SNOM. This advancement not only retains all the functionalities of THz s-SNOM, but also, through optical pump-terahertz probe (OPTP) methods, allows us to access the ultrafast photocarrier dynamics and nanoscale terahertz emissions such as local out-of-plane carrier distributions,^13^ nanoscale ultrafast spin currents.^14^ The most direct application is to employ this advancement to probe the permittivity from the samples, such as the model of the thin-film approximation used in far-field transmission OPTP to obtain the evolution of transient permittivity.^15^ However, the straightforward model in near-field is less developed. The only method currently available for determining the transient permittivity after pumping in Ultrafast THz s-SNOM involves fully recording every time-domain spectrum (TDS) at each time delay and then using FDM or PDM for reverse-solving the permittivity, as shown in Figure 1. This method requires significant time to obtain the complete waveforms and makes numerically solving FDM or PDM computationally expensive. Additionally, this method only tracks the permittivity point-wise, and the relation between different pump delays is still unclear.

In this study, we present a generalized evolutionary model (GEM) applicable to both the PDM and FDM for extracting the permittivity at any time delay from OPTP signals. The principle of our method is based on the assumption that, after excitation, the spectrum within the regime undergoes only multiplicative changes (i.e., the material responds to the pump laser consistently at different frequencies). This assumption adopts a common formalism found in far-field OPTP spectroscopy, where the transient conductivity is decoupled into a time-dependent carrier density term and a frequency-dependent spectral term.^16^ Such an approximation is frequently employed for materials where the relaxation dynamics are primarily governed by carrier recombination rather than rapid variations in carrier mobility or spectral shape.^17^^,^^18^ In the last part, we roughly discuss the application scope of GEM. Although this requirement is rather strict, we demonstrate that our model maintains good correctness and predictive capability under certain approximation conditions that may be applied in fields of quantum and electronic device inspection. Here, to demonstrate the possible application, we selected a set of representative materials widely applied in terahertz technology and possessing distinct ultrafast dynamics: Silicon, GaAs, and Bi_2_Te_3_,^19^ which also trace the evolution from foundational to emerging quantum materials. Additionally, the benefit of our method demonstrates the independence between pump delay and tip oscillation, so that it can be extended to various other s-SNOM models.^20^ The model obeys a source-independent nature, which makes it applicable to ultrafast s-SNOM measurements in various frequency regimes.

## Results and discussion

### Ultrafast terahertz scattering-type scanning near-field optical microscopy and optical pump-terahertz probe signals

**Figure 2:**
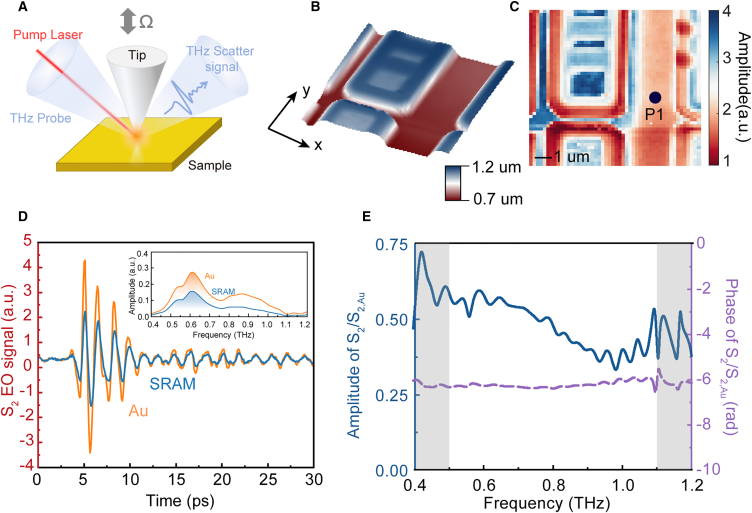
The configuration of ultrafast THz s-SNOM and static TDS of SRAM (A) The configuration of ultrafast THz s-SNOM. (B) AFM image of SRAM. (C) Corresponding THz amplitude scanning image. (D) The time-domain waveform of SRAM and Au, the inset is the spectra of SRAM and Au. (E)The spectrum of SRAM referenced to the spectrum of Au.

Regardless of the frequency regime, the model of s-SNOM involves using an AFM tip to continuously tap the material surface in a harmonic motion, as illustrated in Figure 2A. Under illumination from a light source, the tip-sample-light interaction occurs, allowing us to extract nanoscale optical information through Fourier expansion (More detail about the setup and the principle of the ultrafast s-SNOM is shown in Supplemental Document Methods S1. In our study, we introduced an additional high-photon-energy pump beam into the THz s-SNOM, thereby achieving an ultrafast THz s-SNOM. Upon exposure to the pump laser, the THz probe can be strongly modified to reflect the transient change of conductivity in material.^21^ For OPTP measurement, we fix the probe delay at the peak of the electric field, and the signal is acquired by a photoconductive antenna. We realized that the significant time consumption required to solve the complete permittivity spectrum stems from the inverse solving of the Fourier expansion. The most commonly used numerical methods in s-SNOM involve either directly using a Taylor expansion to solve^22^ or iteratively solving from a pre-assumed spectrum.^23^^,^^24^ Although these two methods yield consistent results, both require substantial computational resources. It is notable that the pump delay *τ* and tip oscillation time *t*. are independent. Assuming that the spectral response of material after optical excitation undergoes only multiplicative changes, we can choose a scattering signal spectrum *S*_*n*_(*ω*) at a specific time as the reference spectrum and normalize it with Au: *η*_*n*_(*ω*) = *S*_*n*_/*S*_*n*,*Au*_(*ω*). The measured OPTP signal is then normalized to the maximum value of the reference signal and denoted as *OPTP*(*τ*). The normalized spectral response at other delay can be denoted as *A*_*n*_(*τ*,*ω*) ≈ *η*_*n*_(*ω*)*OPTP*(*τ*). Using this expression, we can use the FDM and PDM models to calculate the quasi-static scattering coefficient $\beta \left(\omega \right)=\frac{\varepsilon \left(\omega \right)-1}{\varepsilon \left(\omega \right)+1}$ at any given time, thereby obtaining the permittivity *ε*(*ω*). The calculation steps are briefly described as follows: First, the permittivity or quasi-static scattering coefficient *β*(*ω*) of the reference signal is calculated by PDM or FDM. Then, the relationship between *β*′(*ω*,*τ*) and *β*(*ω*) at any time is obtained through Equation 4. Finally, the permittivity at any time is solved by the relationship between *β*′(*ω*,*τ*) and permittivity *ε*(*ω*,*τ*).

### Finite dipole model

For the detailed discussion, the quasi-static scattering coefficient corresponding to the reference frequency spectrum can be obtained by reverse-solving the FDM. According to the FDM model, the following relationships exist between the near-field signal *η*_*n*_(*ω*) and the quasi-static scattering coefficient *β*(*ω*):

(Equation 1) $$\eta_{n}\left(\omega \right)∼\frac{\alpha_{eff,n}\left(\omega \right)}{\alpha_{eff,n,ref}\left(\omega \right)}$$

(Equation 2) $$\alpha_{eff,n}=F_{n}\left(1+\frac{f_{0}\left(H\left(t\right)\right)\beta \left(\omega \right)}{2\left(1-f_{1}\left(H\left(t\right)\right)\beta \left(\omega \right)\right)}\right)$$

Here, “ref” represents the standard sample Au. Among them, *α*_*eff*_ is the effective polarizability, and *f*_0_,*f*_1_ are only related to the geometry of the tip and the distance *H*(*t*) = *z*_*tip*_+*Acos*Ω*t* (where tip oscillation time *t* is the instantaneous time variable of the tip oscillation, and the specific expression is in the Supplemental Document Methods S2. Here, *F*_*n*_ represents the *n*^*th*^ Fourier component at the modulation frequency Ω. For *A*_*n*_(*τ*,*ω*),we can also obtain its quasi-static scattering coefficient *β*′(*ω*,*τ*) by the FDM as follows:

(Equation 3) $$A_{n}\left(\tau ,\omega \right)=\eta_{n}\left(\omega \right)OPTP\left(\tau \right)∼\frac{\alpha_{eff,n,\tau }\left(\omega \right)}{\alpha_{eff,n,ref}\left(\omega \right)}$$

By combining Equation 1 and Equation 3, we can conduct the core relationship of GEM:

(Equation 4) $$\frac{\alpha_{eff,n,\tau }\left(\omega \right)}{OPTP\left(\tau \right)}=\alpha_{eff,n}\left(\omega \right)$$

Equation 4 establishes a direct link between the static state and the pumped state. Substituting the FDM expression (Equation 2) into Equation 4, we derive the relation between the transient *β*′(*ω*,*τ*) and the static reference *β*(*ω*,*τ*) in the Fourier domain:

(Equation 5) $$\alpha_{eff,n,\tau }\left(\omega \right)=F_{n}\left(1+\frac{f_{0}\left(H\left(t\right)\right)\beta^{\prime }\left(\omega ,\tau \right)}{2\left(1-f_{1}\left(H\left(t\right)\right)\beta^{\prime }\left(\omega ,\tau \right)\right)}\right)=OPTP\left(\tau \right)F_{n}\left(1+\frac{f_{0}\left(H\left(t\right)\right)\beta \left(\omega ,\tau \right)}{2\left(1-f_{1}\left(H\left(t\right)\right)\beta \left(\omega ,\tau \right)\right)}\right)$$

By the independence of tip oscillation and pump time delay, we can attempt to retrieve the expanded function from this harmonic relationship. However, a mathematical challenge arises: the s-SNOM signal is demodulated at higher harmonics (*n* ≥ 2) to suppress the far-field background. This high-pass filtering process effectively removes the constant term (DC component) of the time-domain signal. Consequently, the inverse operation from Equation 5 is not unique regarding the constant term. To recover the time-domain form, we must introduce an assumption to restore this lost DC component.

This leads to several possible analytical reconstruction forms. First, if we assume the system response follows a direct multiplicative scaling (which implies restoring the DC constant to 1), we obtain Equation 6:

(Equation 6) $$1+\frac{f_{0}\left(H\left(t\right)\right)\beta^{\prime }\left(\omega ,\tau \right)}{2\left(1-f_{1}\left(H\left(t\right)\right)\beta^{\prime }\left(\omega ,\tau \right)\right)}=OPTP\left(\tau \right)\times \left(1+\frac{f_{0}\left(H\left(t\right)\right)\beta \left(\omega ,\tau \right)}{2\left(1-f_{1}\left(H\left(t\right)\right)\beta \left(\omega ,\tau \right)\right)}\right)$$

Alternatively, if we consider the change in response as a differential quantity (which implies the DC constant term cancels out to 0), we derive Equation 7:

(Equation 7) $$OPTP\left(\tau \right)\frac{f_{0}\left(H\left(t\right)\right)\beta \left(\omega \right)}{1-f_{1}\left(H\left(t\right)\right)\beta \left(\omega \right)}=\frac{f_{0}\left(H\left(t\right)\right)\beta^{\prime }\left(\omega ,\tau \right)}{1-f_{1}\left(H\left(t\right)\right)\beta^{\prime }\left(\omega ,\tau \right)}$$

To generalize Equation 6, we can introduce an arbitrary constant *q* to represent the undetermined DC term, resulting in Equation 8

(Equation 8) $$1+\frac{f_{0}\left(H\left(t\right)\right)\beta^{\prime }\left(\omega ,\tau \right)}{2\left(1-f_{1}\left(H\left(t\right)\right)\beta^{\prime }\left(\omega ,\tau \right)\right)}=OPTP\left(\tau \right)\times (q+\frac{f_{0}\left(H\left(t\right)\right)\beta \left(\omega \right)}{2\left(1-f_{1}\left(H\left(t\right)\right)\beta \left(\omega \right)\right)})$$

It is crucial to note that performing the *n*^*th*^ order Fourier expansion on Equations 6 and 7, or 8 yields the identical harmonic result as Equation 5. Therefore, the optimal form cannot be determined solely by the mathematical derivation but must be validated numerically. Since Equations 6, 7, and 8 are all related to *H*(*t*), to simplify the calculation for solving *β*′(*ω*,*τ*), we can set $t=\frac{\pi }{2}$ in the equations to make ${H\left(t\right)|}_{t=\frac{\pi }{2}}=z_{tip}$ , so that the influence of the tip oscillation can be eliminated.

To demonstrate our method, we sampled scattering signals from the SRAM sample using ultrafast THz s-SNOM in the range of 0.4–1.2 THz. Figure 2B shows the AFM characterization of the SRAM, indicating the presence of multiple distinct islands within our scanning range, with the corresponding amplitude scanning image shown in Figure 2B. The image reveals regions with different scattering characteristics, attributed to different doping concentrations. We fixed the AFM tip at position P1 and measured its scattering signal as depicted in Figure 2E. Our spectral results indicate that our s-SNOM is effective in the range of 0.5–1.1 THz. The spectral results show some fluctuation within the regime, mainly because of the resonant surface waves from the cantilever.^25^

**Figure 3:**
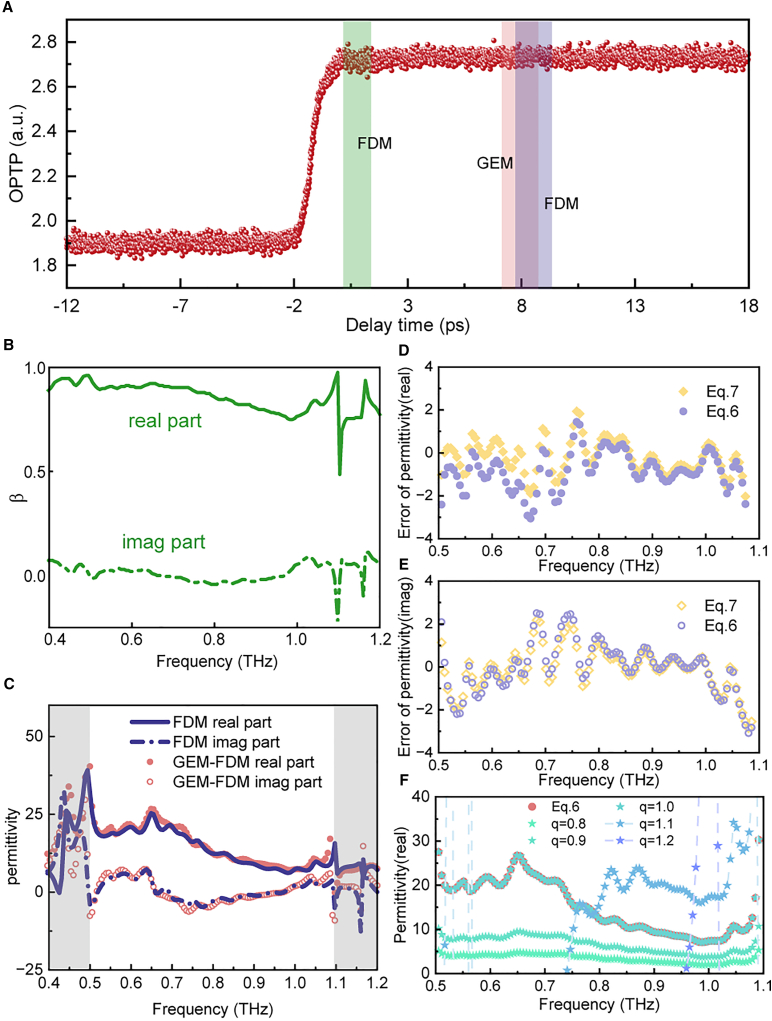
Calculation results based on the FDM model (A) Second-order OPTP signal at P1. (B) The value of *β* calculated by the FDM at the reference time. (C) Permittivity calculated by FDM and our method. (D) Errors of the real part of the permittivity calculated by Equation 6 and Equation 7. (E) Errors of the corresponding imaginary part of the permittivity. (F) Calculation of the permittivity corresponding to different values of *q*.

Fixed at P1, a second-order OPTP signal was traced as shown in Figure 3A. We measured the TDS at 1 ps and 8 ps after laser excitation. We accurately extracted the quasi-static scattering coefficient at 1 ps by an open-source Python package snompy at Figure 3B^26^ Using the reference signal at 1 ps and Equation 6, we also accessed the permittivity at 8 ps, as shown in Figure 3C. These results indicate that our method aligns with the results obtained from the standard FDM within a certain range. Similarly, we also used Equation 7 to extract the permittivity. The real part errors between Equation 6 and FDM are shown in Figure 3D. The mean absolute errors (MAE) of the real part of the permittivity within the effective frequency band are 1.08 and 1.42, respectively. The imaginary part errors of these two formulas are shown in Figure 3E, with the M.A.E. being 0.83 and 0.976. Compared with the results of the real part, the imaginary part errors of the two formulas are close, and both are smaller than the errors of the real part. Based on these two results, for the FDM, we use Equation 7 for calculation. To further validate the reliability of this method, we performed a systematic statistical analysis based on 10 independent repeated measurements. The results confirm that the GEM-derived permittivity falls well within the experimental uncertainty of the conventional FDM. To evaluate the precision and statistical robustness of GEM, we conducted repeated measurements on the SRAM sample. Figure S5 compares the extracted permittivity using both the conventional FDM and GEM with error bands. The overlap between the two methods confirms the accuracy of GEM. Further analysis of the noise characteristics reveals that the average standard deviation for GEM is slightly higher than that of the conventional FDM. This minor increase is attributed to error propagation, as GEM incorporates noise from both the reference TDS and the OPTP signal, whereas the conventional static FDM relies only on the TDS signal. This was further validated by a control test using a fixed OPTP trace, confirming that the additional noise primarily originates from fluctuations in the OPTP signal. Also, we considered different *q* values in Equation 8, with the results shown in Figure 3F. When *q* = 1, Equation 8 degenerates into Equation 6, for *q* < 1, the permittivity decreases while retaining most of the spectral shape. When *q* > 1, the calculated permittivity changes sharply, completely losing the shape of the waveform. Our findings indicate that the value of *q* ought to be 1, under which circumstance Equation 8 degenerates into Equation 6. Therefore, for FDM, we choose Equation 7, which minimizes the error, for calculations.

### Point dipole model

For PDM, Equation 1 and Equation 3 remain unchanged, but the effective polarizability becomes the following formula:

(Equation 9) $$\alpha_{eff,n}=F_{n}\left(\frac{\alpha_{tip}}{1-f\left(H\left(t\right)\right)\beta \left(\omega \right)}\right)$$

Here, *α*_*tip*_, *f*(*H*(*t*)) is defined in the Supplemental Document Methods S3 as well. By applying Equation 4, we can obtain the model corresponding to the PDM as follows:

(Equation 10) $$\beta^{\prime }\left(\omega ,\tau \right)=\frac{1}{f\left(H\left(t\right)\right)}-\frac{1}{OPTP\left(\tau \right)f\left(H\left(t\right)\right)}\left(1-f\left(H\left(t\right)\right)\beta \left(\omega \right)\right){|}_{t=\frac{\pi }{2}}$$

**Figure 4:**
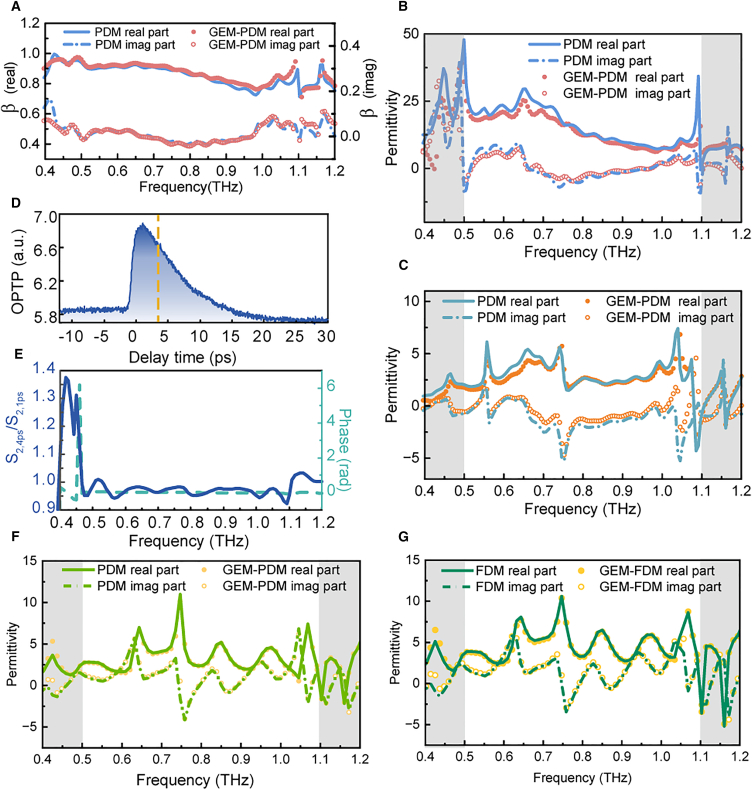
Calculation results based on the PDM model (A) PDM calculation results at the same reference time for P1. (B) Comparison of the permittivity calculated by PDM and our method. (C) Comparison of the permittivity calculation of GaAs after pumping. (D) OPTP signal of Bi_2_Te_3_. (E) Comparison of the transient spectra between the reference delay and the calculation delay. (F) Permittivity of Bi_2_Te_3_ after optical pump excitation. (G)The FDM calculation of Bi_2_Te_3_ at the same time delay.

Similarly, we used Equation 10 and PDM to solve the *β* at 8 ps, as shown in Figure 4A. The permittivity was then calculated, as displayed in Figure 4B. We found that Equation 10 aligns well with the PDM results, further validating the correctness of our formula. Whether using PDM or FDM, we applied the approximation described by *A*_*n*_(*τ*,*ω*)≈*η*_*n*_(*ω*)*OPTP*(*τ*). Next, we demonstrate that our method can predict permittivity within a certain error range. Figure 4C shows the permittivity at an arbitrary delay calculated by PDM and our method for a typical semiconductor GaAs after laser excitation. Also, for the topological insulator Bi_2_Te_3_, we found that our method is also applicable. Its OPTP signal is shown in Figure 4D. The normalized spectrum at 4 ps referenced to the spectrum at 1 ps is illustrated in Figure 4E. Though the amplitude and phase of responses show some fluctuations within the effective frequencies, the calculation results in Figure 4F indicate that the spectral shape of Bi_2_Te_3_ obtained using PDM is largely consistent with our method. Similarly, we calculated the permittivity using the FDM-based GEM (utilizing the optimized Equation 7). As shown in Figure 4G, the results align remarkably well with the standard FDM benchmarks. To validate the temporal consistency of the FDM-based GEM, we also calculated the transient permittivity of Bi_2_Te_3_ at additional pump-probe delays, as shown in Figure S5. The results show excellent agreement between the GEM predictions and the standard static FDM calculations at multiple time points. This demonstrates that GEM exhibits high fidelity in reconstructing the permittivity evolution for both physical models, confirming its versatility as a model-independent acceleration algorithm. The studies of Bi_2_Te_3_ show that despite the strict spectral requirement of our model, our results suggest that our method demonstrates good predictive performance for the permittivity, even within certain spectral fluctuations.

In conclusion, this study presents an analytical method that allows for the prediction of permittivity at any given time from the OPTP signals obtained by ultrafast s-SNOM under certain conditions, thereby significantly enhancing the function of ultrafast s-SNOM in probing permittivity. Our method requires only a reference spectrum and OPTP signal, without the need for full TDS measurements at each time after optical excitation. We demonstrate the applicability of this method in predicting permittivity, quantum, and electronic device inspections by using examples from the ultrafast dynamics of typical SRAM, the semiconductor GaAs, and the topological insulator Bi_2_Te_3_. The analytical results show a remarkable consistency with our proposed model. By correctly applying the optimized reconstruction formula, we demonstrated that GEM accurately reproduces the results of both FDM and PDM. In our experimental setup, the conventional method requires approximately 130 s per data point (including full TDS acquisition and iterative numerical inversion), whereas GEM reduces this processing time to roughly 5 s. This efficiency gain, a reduction factor of over 25-fold, becomes increasingly significant when analyzing complex ultrafast dynamics involving numerous time delays and averaging cycles. The core principle of our model originates from the invariance of the vibration of the tip with respect to pump delay, allowing its generalization to various ultrafast dynamics in the near-field, different tip models, and various frequency bands. Therefore, we expect that our model can be extended to various other s-SNOM models. For instance, the recently developed Quantitative Analytical Spheroid Model^27^ offers an exact analytical solution without relying on phenomenological parameters. Future integration of GEM with this rigorous model holds great promise for achieving high-precision ultrafast permittivity extraction.

### Limitations of the study

In this study, our multiplicative assumption also exists implicitly in many far-field OPTP experiments.^17^ This property holds when the measured material exhibits a purely resistive response (e.g., for the Drude model, the material owns the property with *ωτ*≪1 (*ω* is the frequency, *τ* is the relaxation time)).^18^ In the far-field OPTP experiment, most researchers also track the zero-crossing to gain more accurate dynamics of non-resistive response.^28^ However, the near-field zero-crossing dynamics were only introduced recently^13^ which has already demonstrated remarkable capabilities. Our method can be extended to zero-crossing dynamics as well, with a detailed analysis. Though zero-crossing and OPTP dynamics can realize dynamic extraction of the most material systems in the far-field. There are some eccentric systems such as superconductors that cannot be obtained of their permittivity evolution by only extracting the peak of the THz wave in the far-field because it will distort THz waveform heavily.^29^ This property suggests that our assumption may not hold for materials with bizarre phenomena that will distort THz shape after pumping, such as phonon-polariton interactions, topological transitions, superconductors, and so forth. Specifically, our model assumes that the spectral evolution is dominated by amplitude scaling (multiplicative changes) due to carrier density modulation. It does not account for complex spectral weight transfer, where the spectral shape is fundamentally altered (e.g., shifting from a Drude-like response to a Lorentz-like resonance). In such cases involving significant spectral reshaping, to access the permittivity of these materials, the most accurate method is to record the full spectrum information point-wise (namely the conventional method).

## Resource availability

### Lead contact

Further information and requests for resources and reagents should be directed to and will be fulfilled by the lead contact, Xiaojun Wu (xiaojunwu@buaa.edu.cn).

### Material availability

All samples reported in this article will be shared by the lead contact upon request.

### Data and code availability

• All data reported in this article will be shared by the lead contact upon request.
• All codes used in this article will be shared by the lead contact upon request.
• Any additional information required to reanalyze the data reported in this article is available from the lead contact upon request.

## Acknowledgments

This work is supported by the Scientific Research Innovation Capability Support Project for Young Faculty (ZYGXQNJSKYCXNLZCXM-I3), the 10.13039/501100012166National Key Research and Development Program of China (2022YFA1604402), the 10.13039/501100001809National Natural Science Foundation of China (U23A6002, 92250307), and the 10.13039/501100009592Beijing Municipal Science & Technology Commission, Administrative Commission of Zhongguancun Science Park (Z251100006925005).

## Author contributions

X.W.: conceptualization, funding acquisition, project administration, supervision, and writing – review and editing; Z.Z.: data curation, investigation, and writing – original draft preparation; Z.H.: data curation, investigation, and writing – original draft preparation; A.X.: investigation and writing – review and editing; J.L.: investigation; J.C.: investigation; P.L.: investigation; M.D.: investigation; T.N.: visualization; A.E.M.: investigation.

## Declaration of interests

The authors declare no competing interests.

## STAR★Methods

### Key resources table

**Table:** 

REAGENT or RESOURCE	SOURCE	IDENTIFIER
**Chemicals, peptides, and recombinant proteins**		

Gold (Au) reference	Neaspec	https://www.neaspec.com/
SRAM sample	Neaspec	https://www.neaspec.com/
GaAs sample	Lab made	N/A
Bi2Te3 sample	Lab made	N/A

**Software and algorithms**		

Python	Python Software Foundation	https://www.python.org/
snompy package	Vincent et al.	https://snompy.readthedocs.io/
Origin	OriginLab	https://www.originlab.com/
Gwyddion	Gwyddion	http://gwyddion.net/

**Other**		

AFM Tip	Neaspec	https://www.neaspec.com/

### Method details

The size of our samples is approximately 1.5 cm × 1.5 cm. The experimental setup employs a fiber-coupled optical architecture where temporal synchronization is managed by fiber-optic delay lines integrated within the controller. These lines independently control the transmitter and receiver delays to adjust the THz-TDS delay and the pump-probe delay. In the stage of extracting the equilibrium state, we use the scanning mode to raster-scan the sample for obtaining its morphology and scattering characteristics. The scanning process costs 8 minutes per picture. The integration time is set to 100 ms. For single-point TDS signal extraction, the integration time is set to 300 ms and the averaging time is set to 10. In the stage of obtaining the OPTP signals, we use the same scanning parameters as in the first stage. For single-point OPTP signal extraction, the integration time is set to 100 ms and the averaging time is set to 1. According to FDM and PDM, the scattered signals are normalized by dividing the scattered THz signal of gold. The parameters used for the model calculations are based on the experimental conditions: the tapping amplitude is 240 nm, the tip radius of curvature is 20 nm, the effective tip length is 600 nm, and the empirical geometry factor *g* is set to 0.7×*exp*(0.06*i*).

### Quantification and statistical analysis

To make quantification, we employed Python, Origin and, Gwyddion, and the pre-installed software of SNOM to analyze our data. The PDM and FDM computations were calculated by using an open-source python package snompy.
