# Supplementary material for: A model of optical pump-terahertz probe by ultrafast terahertz near-field microscopy
Source: iScience. 2026 Feb 25;29(3):115145. doi: 10.1016/j.isci.2026.115145 (PMC12991951; doi:10.1016/j.isci.2026.115145)
Supplement: Document S1. Figures S1–S6 — and Methods S1–S3 [file mmc1.pdf]

iScience, Volume *29*

Supplemental information

A model of optical pump-terahertz probe by ultrafast terahertz near-field microscopy

Zijian Zhang, Ziyu Huang, Aojie Xu, Jing Li, Peiyan Li, Jiahua Cai, Mingcong Dai, Tianxiao Nie, Amine El Moutaouakil, and Xiaojun Wu

Methods S1. The setup of ultrafast s-SNOM.

The sketch of the experimental setup is shown in Fig. S1. The laser at a center wavelength of 1560 nm was transformed into 780 nm by second harmonic generator before illuminated the sample. THz wave was generated and detected by photoconductive antenna and the incident THz wave illuminated the sample with an incident angle of 36°. Additional details can be found in[1].

Upon illumination by a light source, a harmonic nanoscale tip-sample-light interaction occurs (described by point dipole model (PDM) and finite dipole model (FDM)). This interaction information is carried by scattered light to the far field, where it is received by the photoconductive antenna. By performing a Fourier expansion on the scattered signal, we obtain high-order information about nanoscale interactions, which is the actual signal we detect in our system. Through inverting the Fourier expansion of the high-order scattered signal and applying PDM or FDM, we can solve for the local permittivity.

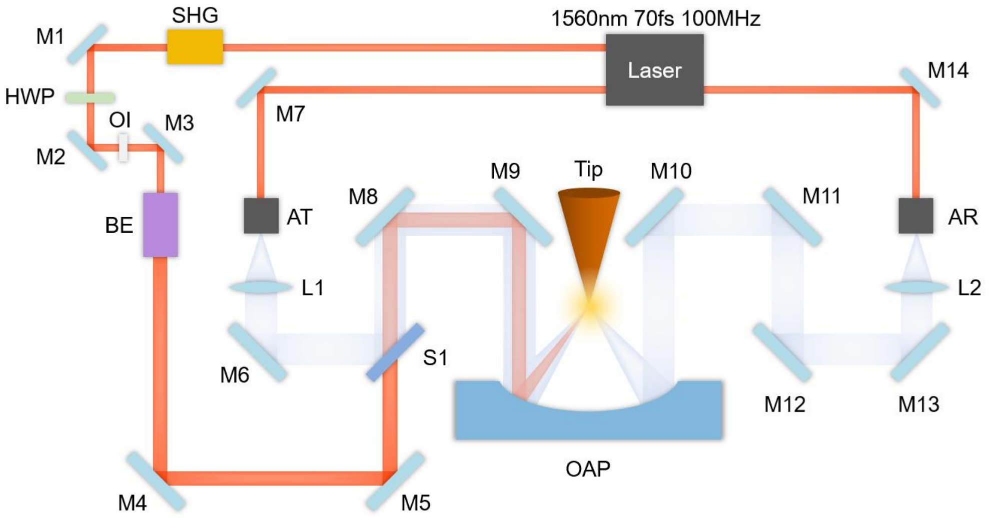

Figure S1 Ultrafast THz s-SNOM. The laser provides 70 fs pulses at 100 MHz, at a center wavelength of 1560 nm. SHG: second harmonic generator, HWP: half wave plate, OI: optical isolator, BE: beam expander, S1: beam splitter, OAP: off-axis parabolic mirror, AT,AR: photoconductive antenna for transmitting and receiving respectively, L1-2 and M1-14: lens and mirrors. Red line stands for laser and blue line stands for THz pulse.

Methods S2. The expression of FDM.

The tip-sample interaction is shown in Fig. S2. In finite dipole model[2], we're treating the tip as an ellipsoid above the surface of sample shown in Fig. S3. The geometry of the tip can be described by the effective length L_{eff} and the radius of the tip r_t.

The effective polarizability α_{eff} in FDM can be written:

$$\alpha_{eff} = 1 + \frac{f_0(H(t))\beta(\omega)}{2(1 - f_1(H(t))\beta(\omega))} \tag{S1}$$

$$f_{0,1} = \left(g - \frac{r_t + 2H + W_{0,1}}{2L_{eff}}\right) \frac{\ln(4L) - \ln(r_t + 4H + 2W_{0,1})}{\ln(4L_{eff}) - \ln r_t} \tag{S2}$$

In Eq. (S2), $g = 0.7\exp(0.06i)$, $H = z_{tip} + A\cos\Omega t$, r_t stands for the radius of tip $W_0 \approx 1.31 r_t$, $W_1 \approx 0.5 r_t$.

During our experiments, the effective length $L_{eff} = 1200 \times 10^{-9}\ m$, the radius of tip $r_t = 100 \times 10^{-9}\ m$, the distance between tip and material $z_{tip} = 10^{-9}\ m$, the amplitude of our AFM tip $A = 240 \times 10^{-9}\ m$, the modulation period $\Omega = 54\ kHz$.

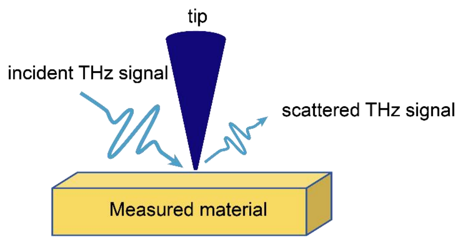

Figure S2 The model of THz s-SNOM

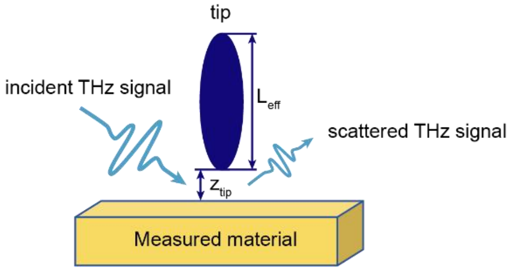

Figure S3 The finite dipole model

Methods S3. The expression of PDM.

In point dipole model[3], the tip is regarded as a dielectric sphere above the surface of sample shown in Fig. S4. The geometry of the tip can be described by the radius of the tip r_t.

The effective polarizability α_{eff} in PDM can be written as:

$$\alpha_{eff} = \frac{\alpha_{tip}}{1 - f(t)\beta(\omega)} \tag{S3}$$

Here, α_{tip} stands for polarizability of the sphere, which is the function of the permittivity ϵ_{tip} and radius r_t of the sphere:

$$\alpha_{tip} = 4\pi r_t^3 \frac{\epsilon_{tip} - 1}{\epsilon_{tip} + 2} \tag{S4}$$

Still, the function $f(t) = \dfrac{\alpha_{tip}}{16\pi(r_t + H(t))^3}$ encapsulates the geometry of the tip.

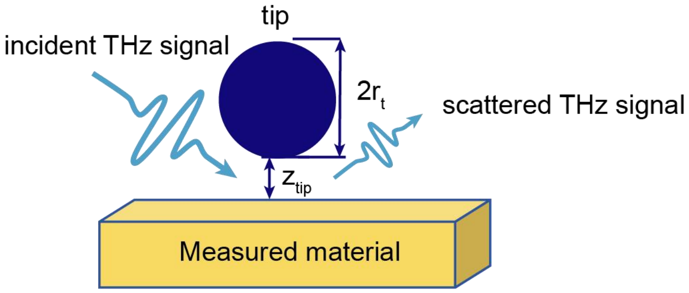

Figure S4 The point dipole model

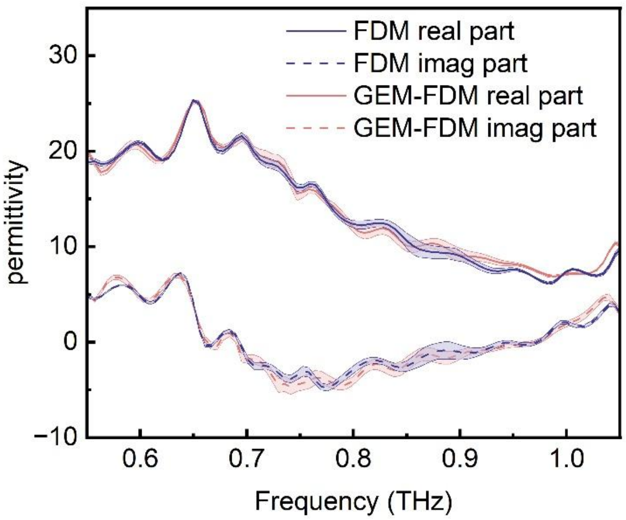

Figure S5 Statistical error analysis for the SRAM sample. The plot compares the extracted permittivity using the conventional FDM (dashed lines) and the GEM (Eq. 7, solid lines) over 10 independent measurements. The shaded error bands represent the standard deviation (σ). The average standard deviation across the effective frequency band is 0.396 for GEM and 0.313 for the conventional FDM.

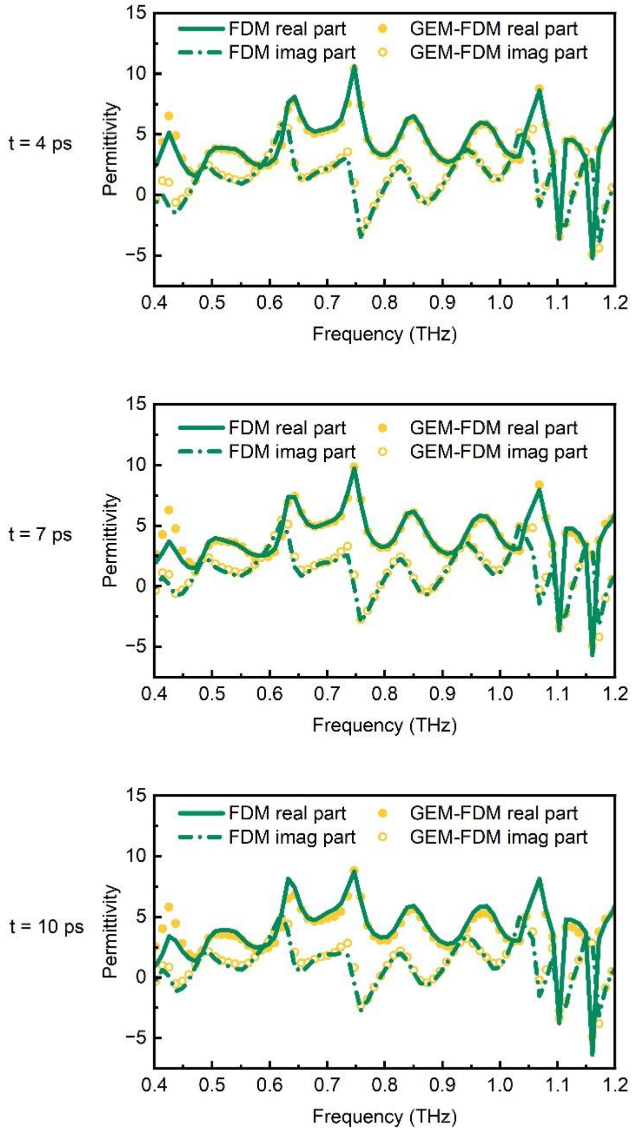

Figure S6. Comparison for Bi_2Te_3 between FDM and GEM-FDM at different pump time delays using Eq. 7.

References

1. Huang, Z., Li, J., Li, P., Du, L., Dai, M., Cai, J., Ren, Z., Nie, T., and Wu, X. (2025). Nanoscale ultrafast dynamics in Bi2Te3 thin film by terahertz scanning near-field nanoscopy. iScience *28*, 111840. https://doi.org/10.1016/j.isci.2025.111840.

2. Cvitkovic, A., Ocelic, N., and Hillenbrand, R. (2007). Analytical model for quantitative prediction of material contrasts in scattering-type near-field optical microscopy. Optics express *15*, 8550–8565. https://doi.org/10.1364/OE.15.008550.

3. Vincent, T., Liu, X., Johnson, D., Mester, L., Huang, N., Kazakova, O., Hillenbrand, R., and Boland, J.L. (2024). snompy: a package for modelling scattering-type scanning near-field optical microscopy. arXiv preprint arXiv:2405.20948.
